# A Lightweight Track Feature Detection Algorithm Based on Element Multiplication and Extended Path Aggregation Networks

**DOI:** 10.3390/s25185753

**Published:** 2025-09-16

**Authors:** Hong Qiu, Dayong Yang, Juanhua Cao, Jingqiang Ming, Kun Jiang, Weijun Wu

**Affiliations:** 1 School of Advanced Manufacturing, Nanchang University, Nanchang 330031, China; 2 Jiangxi Technical College of Manufacturing, Nanchang 330031, China; 3 Jiangxi Everbright Measurement and Control Technology Co., Ltd., Nanchang 330031, China

**Keywords:** track inspection, YOLO11, elemental multiplication, lightweight network

## Abstract

Aiming at the problems of excessive computational load, insufficient real-time performance, and an excessive amount of model parameters in track inspection, this paper proposes a lightweight track feature detection module (YOLO-LWTD) based on YOLO11n: first, the StarNet module is integrated into the backbone network, and its elemental multiplication operation is utilized to enhance the feature characterization capability; second, in the neck part, a lightweight extended path aggregation network reconstructs the feature pyramid information flow paths by combining with the C3K2-Light module to enhance the efficiency of the multi-scale feature fusion; finally, in the head part, a lighter and more efficient detection header, Detect-LADH, is used to reduce the feature decoding complexity. Experimental validation showed that the improved model outperforms the benchmark model in precision, recall, and mean average precision (MAP) by 0.5%, 2.0%, and 0.8%, respectively, with an inference speed of 163 FPS (a 38.1% improvement). The model volume is compressed to 1.5 MB (a 71.1% lightweight rate). This provides an energy-efficient solution for lightweight track detection tasks geared towards embedded deployment or real-time processing.

## 1. Introduction

As the demand for intelligent operation and maintenance of rail transportation continues to increase, the importance of track inspection is becoming increasingly prominent. The track inspection system describes the geometric position parameters of the track with the running mileage as the independent variable; however, under the influence of cumulative error, the mileage of the geometric deviation position marking in long-distance measurement may have a significant deviation from the actual position. To systematically monitor the condition of key components, such as sleepers, fasteners, roadbeds, and turnouts, the detection accuracy of track inspection is significantly constrained by the cumulative mileage error, which directly leads to deviations in the condition assessment and spatial localization inaccuracy. It significantly affects the reliability of decision-making regarding operation and maintenance [1].

To achieve dynamic error correction, a real-time error compensation mechanism based on feature matching can be established by detecting features with fixed patterns in the track as mileage calibration points [2,3]. Therefore, high-precision track feature detection technology has become the core link for improving the robustness of the intelligent inspection system, which is of key significance in guaranteeing the safe operation and maintenance of the entire track life cycle.

In recent years, researchers have used various sensors and optimization algorithms to extract features from tracks for research on track feature detection [4]. Wang [5] used an odometer to detect the speed of the train on the track and the distance traveled, which is still limited due to the acceleration and deceleration skidding of the train and the wear and tear of the wheels; Wei [6] introduced light detection and ranging (LIDAR) equipment to detect the track plane and used the moving average filter (MAF) algorithm to detect and classify the track, but the method is too computationally intensive and it has very high requirements for the hardware terminal; Wang et al. [7] fused the radar-acquired multi-cycle tunnel profile point cloud data through the localization algorithm and used the subway tunnel modeling algorithm to establish a standard tunnel profile model to process the fused data; Zhang [8] used the existing communication optical fiber along the railroad line to detect the track plane and classify the track and proposed an interferometric technique based on Rayleigh backscattered signals in optical fibers for identification and localization of railroad vehicles using existing communication cables along the railroad, but the method still has some limitations; Olaby [9] proposed a method for railroad localization by using RFID technology to align vehicles to the location of turnouts and crossings on the railroad network; Lian [10] proposed a new modular visual processing framework based on the multi-target tracking module of dynamic regions of interest to assign a unique identification code to each landmark for continuous train localization; Spinsante [11] proposed a hybrid GNSS method for train localization, but the method has certain requirements for GNSS signals; Qin [12] proposed a new method using data fusion techniques with mileage-corrected track geometry inspection data combined with the uncorrected velocity information of axlebox acceleration inspection data to correct the mileage deviation of axlebox acceleration inspection data; and Chen [13] proposed an on-board railroad positioning system assisted by digital track maps using the Jetlink inertial navigation system (SINS) and OD, which effectively suppresses the accumulation of the train’s position error. In summary, the current track detection method relies on high-precision equipment to achieve accurate track detection, and the error caused by this equipment also requires higher algorithm complexity. At the same time, the detection model needs to be deployed in the mobile edge device, and its resource constraints and multi-threaded environment also require the model to have a smaller number of parameters. Therefore, lightweight, efficient, and accurate track feature detection technology has become crucial in ensuring line safety and operational and maintenance efficiency.

The rapid development of deep learning technology has injected unprecedented vitality into the field of computer vision, and vision-based track inspection is gradually becoming a key research direction [14]. Phaphuangwittayakul [15] utilized the Dual Attention Visual Transformer (DaViT) to construct RailTrack-DaViT, effectively capturing both global and local information to achieve accurate track detection; Xiao [16] developed a novel fusion model combining the Segment Anything Model and U-Net network to perform detailed identification and segmentation of track scaling areas; Bottalico [17] developed a method based on 3D vision to identify inherent features already present on track structures; HU [18] enhanced the detection in complex slab track scenarios using synthetic images based on the YOLO architecture; Ma [19] improved the YOLOv8 algorithm for detecting train track fasteners, achieving good detection results; Luo [20] automated ballast detection using computer vision methods, employing BSV to thoroughly assess continuous track sections; Shen [21] improved YOLOv7 and Center-Point for detecting visible light images and point clouds, respectively, and used AED as a new metric in the data correlation module to track detection results between images and point clouds, effectively enhancing the correlation robustness and reducing the tracking errors. In terms of algorithm-based applications, Xia [22] proposed an Odess iteration, significantly reducing the computational overhead of similarity detection while achieving a high detection accuracy and high compression ratios, while Zou [23] proposed a novel management-friendly duplicate data deletion framework named MFDedup, maximizing the locality; these methods open new horizons for embedded deployment in track detection applications.

Based on the current research status and existing problems, this paper proposes a lightweight track feature detection algorithm, YOLO-LWTD, for track inspection tasks, building upon YOLO11 [24]. First, StarNet is used to replace the original backbone network, significantly reducing the model complexity. Second, the feature fusion network is reconfigured to be lightweight, and the efficient C3K2-Light module is introduced. Finally, the model’s performance is further enhanced by the detection head structure. The structure further enhances the model’s detection performance.

The structure of this paper is as follows: Section 1 provides a summary of the current state of research; Section 2 systematically describes the overall architecture of the track detection algorithm based on the improved YOLO11 and the design principle of its optimization module; Section 3 outlines the data acquisition and model-training process; Section 4 analyzes the differences in the performance indexes between the proposed algorithm and the main benchmark methods through comparative experiments and systematically evaluates the experimental results; and Section 5 summarizes the entire paper and presents constructive perspectives for future research directions.

## 2. Proposed Methodology

### 2.1. YOLO11

**Figure 1:**
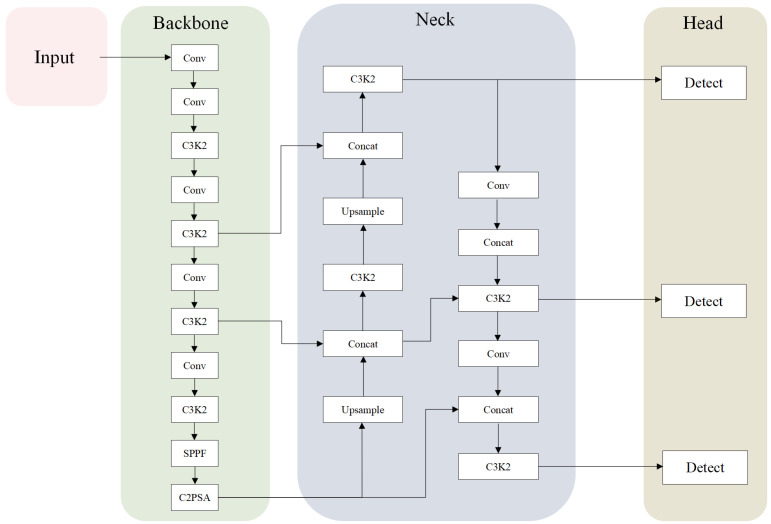
YOLO11 network architecture.

The YOLO (You Only Look Once) series of algorithms, a representative research algorithm in the field of target detection, employs an end-to-end single-stage detection architecture, which enables the efficient detection and precise localization of multiple target objects in images. YOLO11 is the latest model proposed by the Ultralytics team, featuring main innovations that include the introduction of the C3K2 module to optimize the shallow feature extraction process, the incorporation of the C2PSA attention mechanism to enhance feature capture, and the addition of depth-separable convolution (DWConv) to the detection head. In terms of the model architecture, YOLO11 consists of three core components: a feature extraction backbone network (backbone), a multi-scale feature fusion neck network (neck), and a target detection head (head); its overall architecture is shown in Figure 1.

YOLO11 mainly consists of three parts: backbone, neck, and head. The backbone network part of YOLO11 is used to extract the multi-scale feature maps of the input image. It includes modules such as Conv, C3K2, SPPF, and C2PSA. C3K2 enhances the overall performance of feature extraction. In contrast, the spatial attention (C2PSA) module is combined with the SPPF, which enables the model to adaptively focus on the salient regions in the image to enhance the key feature expression ability; the neck network adopts a combined bi-directional feature fusion structure (PANet) that combines FPN and PAN, in which the C3K2 module fuses features at different scales more efficiently; the detection head part follows the decoupled head of YOLOv8, but YOLO11 adds two depth-separable convolutions (DW-Conv) to the classification detection head to substantially reduce the computation amount without losing accuracy and, at the same time, significantly reduces the computational effort. For the regression loss, a composite loss function combining distribution focal loss and CIoU (complete intersection over union) is used; for the classification loss, distribution focal loss (DFL) is used for the optimization, which adaptively adjusts the weights of positive and negative samples, effectively alleviating the category imbalance problem [25].

YOLO11 provides five models with different network depths and widths—n (nano), s (small), m (medium), l (large), and x (extra-large)—based on the synergistic tuning of the network depths and widths, as well as the structural parameter counts of each model variant under the condition of an input resolution of 640 × 640 pixels (parameters) and floating point operations (FLOPs) metrics for each model variant at an input resolution of 640 × 640 pixels are shown in Table 1.

### 2.2. YOLO-LWTD

Aiming to enhance the feature extraction efficiency and meet the requirements for lightweight models in multi-target detection tasks for tracking scenes, this study improves the model by utilizing YOLO11n as the base network and proposes a new lightweight track feature detection model. The innovative improvements of the model are mainly reflected in the following three aspects:

- In the backbone feature extraction part, the element-level feature interaction mechanism unique to StarNet is innovatively introduced to enhance the feature extraction;
- In the neck part of the model, a lightweight extended path aggregation network and the C3K2-Light module are adopted to achieve efficient fusion of multi-scale features by optimizing the information propagation path of the feature pyramid;
- In the head part, a lighter and more efficient detection head Detect-LADH is adopted, and this structure significantly reduces the computational complexity by simplifying the feature-decoding process while ensuring the detection accuracy.

**Figure 2:**
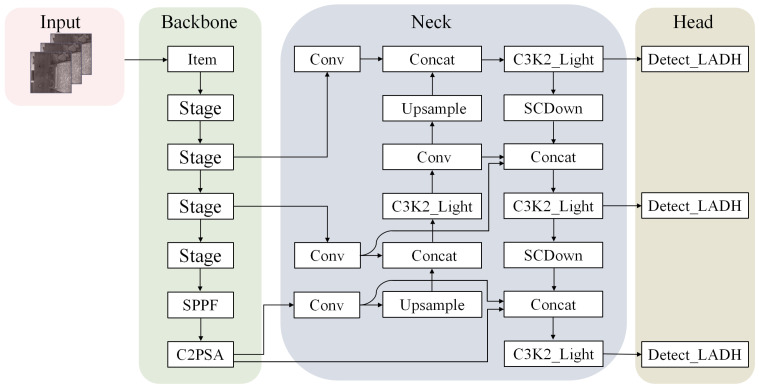
YOLO-LWTD network architecture.

The above improved complete network structure is shown in Figure 2.

### 2.3. StarNet

The backbone network architecture of YOLO11 constructs a multilevel feature extraction system by integrating components such as the C3K2 module, SPPF, C2PSA, and the product layer. Although this module combination strategy effectively improves the feature expression capability of the network, it also significantly increases the amount of parameter computation required by the model, resulting in a decrease in the inference speed and negatively impacting the deployment efficiency of the model in real-world application scenarios. To effectively address the performance bottleneck mentioned above, this paper proposes a backbone network reconfiguration scheme based on the StarNet network.

StarNet [26] is an efficient neural network architecture based on elementary multiplication operations. Unlike the standard linear dot product operation, it employs element-wise multiplication to construct a mapping relation from a low-dimensional feature space to a high-dimensional, nonlinear space. This operation does not increase the width of the network, and not only preserves the local specificity of the input features but also significantly improves the discriminative ability of the model through nonlinear interactions. For target detection networks, such as YOLO, the elemental multiplication operation is particularly suitable for capturing visual features with subtle structural differences.

In a single-layer neural network, the neuron inputs can be represented by the representation of the multiplication operation as in Equation (1), where ⋆ represents the multiplication operation, *W* represents the weights of the input neurons, and *B* represents the bias of the input neurons.

(1) $$\left(W_{1}^{T}X+B_{1}\right)⋆\left(W_{2}^{T}X+B_{2}\right)$$

Based on Equation (1), the weight matrix and bias are then combined into a single entity, denoted as $W=\left[\begin{array}{c} W\\ B \end{array}\right]$, corresponding to $X=\left[\begin{array}{c} W\\ 1 \end{array}\right]$, and the fusion of the characteristics of two linear transformations is expressed by element multiplication, resulting in a single output channel conversion and a single-element-input element multiplication, as shown in Equation (2). This equation defines $W_{1},W_{2},X\in R^{(d+1)\times 1}$, where *d* is the input channel number; it can also be extended to the case of multiple output channels and processing of multiple feature elements, i.e., $W_{1},W_{2}\in R^{\left(d+1\right)\times \left(d^{\prime }+1\right)}$, where $X\in R^{(d+1)\times n}$. Meanwhile, *i* and *j* are used to index the channels and α is the coefficient of each item, where the expression for the coefficients is shown in Equation (3).

(2) $$\begin{array}{cc} W_{1}^{T}X⋆W_{2}^{T}X\; & =\left(\sum_{i=1}^{d+1}W_{1}^{i}X^{i}\right)⋆\left(\sum_{j=1}^{d+1}W_{2}^{j}X^{j}\right)\\ \; & =\sum_{i=1}^{d+1}\sum_{j=1}^{d+1}W_{1}^{i}W_{2}^{j}W^{i}X^{j}\\ \; & =\underset{(d+2)(d+1)/2\;\mathrm{items}}{\underset{︸}{\alpha_{\left(1,1\right)}X^{1}X^{1}+\cdots +\alpha_{\left(4,5\right)}X^{4}X^{5}+\cdots +\alpha_{\left(d+1,d+1\right)}X^{d+1}X^{d+1}}} \end{array}$$

(3) α(i,j)=0w1iw2jifi=j,w1iw2j+w1jw2iifi!=j.

From Equation (3), it can be seen that except for the term $\alpha_{\left(d+1,:\right)}x^{\left(d+1\right)}x$, the rest of the terms all exhibit linear irrelevance to the term *x*, i.e., they are all independent dimensions. Therefore, the element multiplication operation in the *d*-dimensional space yields a representation in the $\frac{\left(d\;+\;2)(d\;+\;1\right)}{2}\approx {\left(\frac{d}{\sqrt{2}}\right)}^{2}$ dimensional space, which significantly enhances the dimensionality of the features. This computational mechanism strikes a good balance between computational complexity and model expressiveness, effectively retaining and extracting rich deep semantic information even under low-resolution input conditions, making it particularly suitable for real-time inspection tasks in track or near-track.

**Figure 3:**
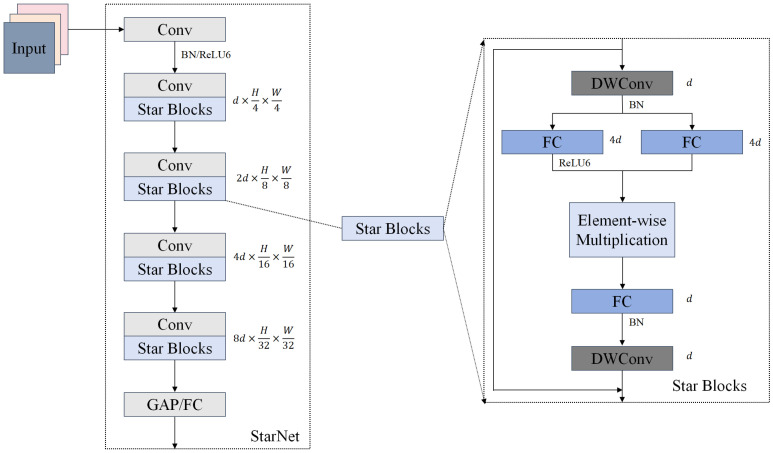
StarNet network architecture.

The network structure of StarNet, based on the above elemental multiplication, is shown in Figure 3. It employs an efficient four-stage hierarchical feature extraction framework that achieves progressive expansion of feature dimensions by multiplying the number of channels stage by stage. Specifically, the network first performs basic feature extraction on the input image through the first convolutional layer, followed by deep feature extraction through four-layered architectures. A convolutional layer downsamples each layered architecture, and feature extraction is performed using the StarBlocks module, which consists of two deeply divisible convolutions and three fully connected networks. First, batch normalization is introduced after deep convolution to facilitate information fusion, and batch normalization can improve the computational efficiency of the model; subsequently, the result of batch normalization is passed through the ReLU6 activation function to introduce the nonlinear transformation capability; after this, a new high-dimensional feature space is generated through the elemental multiplication operation; then, the result of the operation is passed through the fully connected layer to integrate the features for preprocessing of the classification task; and, finally, the features are efficiently fused by deep convolution at the end of StarBlocks to further enhance the feature extraction capability. StarNet abandons the traditional method of expanding the network width (i.e., increasing the number of channels) to enhance the expression capability of the model and realizes high-dimensional feature mapping in low-dimensional space, which not only significantly improves the expression capability of the model but also improves the performance of the model, which can also be used for classification tasks. This not only significantly improves the efficiency of the feature extraction and characterization ability but also significantly reduces the computational complexity, achieving the design goal of model lightweighting.

In YOLO-LWTD, StarNet serves as the front-end component of the backbone, performing basic feature extraction on the input track images to obtain multi-scale feature information. Specifically, the first convolutional layer of StarNet corresponds to the item module in the backbone shown in Figure 2. Subsequent convolutional layers and Star Blocks are alternately stacked to form four stage modules. Finally, SPPF and C2PSA perform additional processing on low-scale features to enhance the model’s ability to express low-frequency features in images.

### 2.4. EPAN

**Figure 4:**
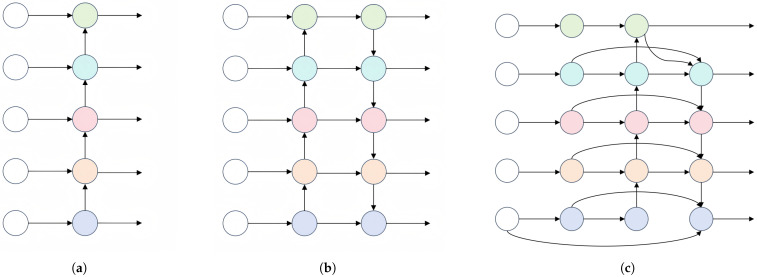
Diagram of the three neck network structures: (**a**) FPN; (**b**) PANet; (**c**) EPAN.

YOLO11 adopts the path aggregation network (PANet) as its neck part of the pyramid structure, as shown in Figure 4b. Compared with the traditional Feature Pyramid Network (FPN) shown in Figure 4a, PANet adds a bottom-up pathway, where this two-way feature fusion strategy realizes the efficient transfer and fusion of high-level feature information to the low-level, which not only makes the feature map rich in semantic information and precise location information but also breaks through the limitation of unidirectional information flow in the FPN and effectively alleviates the problem of shallow feature information loss. Meanwhile, the feature information is distributed among layers according to the network size, with smaller features assigned to lower layers and larger features to higher layers, thereby optimizing the utilization of multi-scale features. Thanks to this structure, YOLO11 can detect the scale, shape, and class of the target more accurately, while the model’s characterization ability is further enhanced by gradually increasing the depth and resolution of the feature map.

However, in practical applications for track detection, YOLO11 shows certain shortcomings in detection. The primary reason is the unsatisfactory effect of feature fusion, which is insufficient for integrating low-level features (e.g., detailed information about the target) and high-level features (e.g., global context information), resulting in limited accuracy and recall in target detection. Therefore, for tracking images with variable background information and irregular image noise, PANet still has limitations in capturing the detailed features of the image, which significantly affects the model’s performance in complex scenes [27].

To address these limitations, this paper further optimizes the feature fusion mechanism based on the architecture of PANet, achieving finer multi-scale feature interactions by introducing an efficient extended path aggregation network (EPAN). As shown in Figure 4c, EPAN’s front end introduces additional feature-processing modules to perform more refined processing of the main features, making them better suited to the complexity of track scenes and addressing the shortcomings of traditional multi-scale fusion networks in terms of feature depth information mining. Meanwhile, an innovative cross-layer jump connection, similar to a residual structure, has been introduced to enhance the retention and utilization of spatial detail information, enabling the model to more effectively capture the key features of orbital scenes. Additionally, by optimizing the information flow path, the model achieves its lightweighting goal by carrying richer effective information with fewer feature layers.

**Figure 5:**
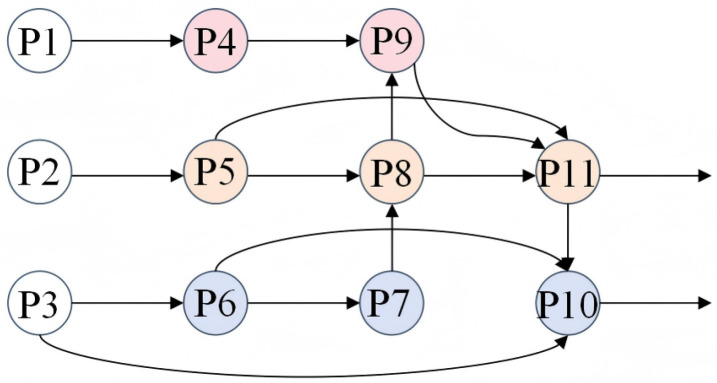
Feature relationship diagram.

In Figure 4c, the feature maps of each row in EPAN have the same scale, but there are differences in how each feature map is processed in detail. The specific implementation process is described as follows: First, three feature maps of different scales—P1, P2, and P3—are extracted from the backbone network. Next, the feature maps P1, P2, and P3 output by the backbone network are processed through a 1 × 1 convolution module to generate P4, P5, and P6, respectively, aiming to achieve a nonlinear mapping between the input channels. Subsequently, P6 is upsampled to generate P7; P7 is concatenated with P5, then processed through the C3K2-Light module and a convolutional layer to generate P8; P8 is then concatenated with P4 and fed into the C3K2-Light module for processing to generate P9. Finally, P9 is downsampled and concatenated with P8 and P5, then processed through the C3K2-Light module to generate P11; and P11 is downsampled and concatenated with P3 and P6, then processed through the C3K2-Light module to generate P10. Ultimately, the feature maps P9, P10, and P11 are output from EPAN and fed into the object detection head. The correspondence between feature maps is shown in Figure 5.

### 2.5. C3K2-Light

**Figure 6:**
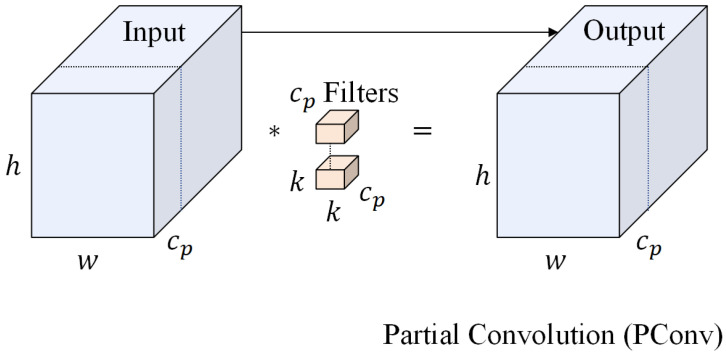
PConv network architecture.

To construct a more lightweight YOLO11 detection network and achieve the optimal balance between model efficiency and detection accuracy while ensuring track detection performance, this study is inspired by FasterNet and improves the C3K2 module in YOLO11 based on Partial Convolution (PConv [28]), which makes clever use of the redundancy of channels in the feature map to extract spatial features while keeping other channels undisturbed. Channel redundancy is maintained in the feature map to perform traditional convolution operations on only some of the input channels to extract spatial features while keeping the other channels undisturbed, and this selective computation mechanism significantly reduces the amount of floating-point operations (FLOPs). The structure of PConv is shown in Figure 6.

For regular convolution with input $X\in R^{c\times h\times w}$ and output $Y\in R^{c\times h\times w}$, the FLOPs are shown in Equation (4), where *c* is the channel number, *h* and *w* are the height and width of the input data, and k is the size of the convolution kernel. Furthermore, the FLOPs for PConv are shown in Equation (5), where $c_{p}$ is denoted as the channel number. Since PConv performs conventional convolution operations only on the first and last consecutive channels of the input feature map while keeping the middle channels unchanged, this selective computational strategy makes its FLOPs significantly lower than that of the conventional convolution method and significantly reduces the overall parameter count of the model, thus realizing the balance between the computational efficiency and the feature expression capability.

(4) $$h\times w\times k^{2}\times c^{2}$$

(5) $$h\times w\times k^{2}\times c_{p}^{2}$$

**Figure 7:**
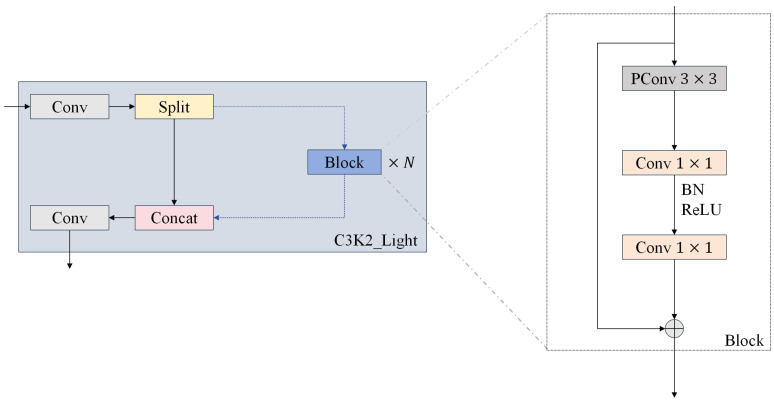
C3K2-Light network architecture.

We designed the C3K2-Light module, shown in Figure 7, based on PConv to optimize the balance between detection accuracy and computational efficiency. The core architecture of the module employs a 3×3 PConv as the central layer, which not only retains the attention property of standard convolution in the center region of the sensory field but also significantly reduces the computational complexity through a selective channel computation strategy. To enhance the feature characterization capability, the module cascades two 1×1 convolutional layers after the PConv layer, extending the receptive field. It then fuses the features of the PConv and the regular convolution through residual concatenation, ensuring feature diversity while facilitating fast inference. In particular, the module places BatchNormalization after the intermediate convolutional layer and supplements it with the ReLU activation function, which not only accelerates the model’s convergence but also significantly improves its inference efficiency.

### 2.6. Detect-LADH

The decoupled two-branch detection head structure adopted by YOLO11 improves the task specificity by independently processing classification and regression tasks. However, this architectural design has three significant drawbacks: first, the multiple convolutional operations in the two branches significantly increase the number of model parameters and computational complexity, making it difficult to achieve efficient deployment on low-computing-power devices or meet real-time detection requirements; second, since the feature processing of the classification and regression branches is completely isolated, the network cannot effectively utilize the complementary high-level semantic features extracted by the backbone network, thereby limiting the model’s representational capability and detection performance in track inspection tasks; additionally, the original decoupled head uses the same convolutional layer at the top of the network for both regression and classification, but these tasks have different focuses, leading to potential conflicts during the detection process.

**Figure 8:**
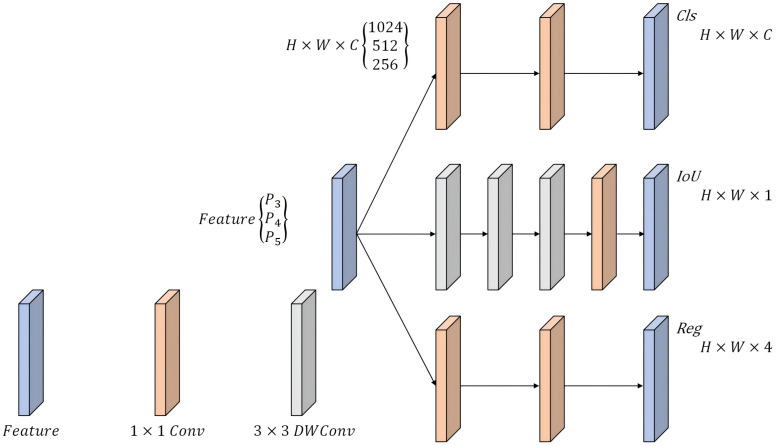
Detect-LADH network architecture diagram.

We introduce a lightweight asymmetric detection head (LADH [29]) to address the above issues; the structure is illustrated in Figure 8. This architecture is based on a task-driven design philosophy and uses a three-channel separated network to handle the classification, regression, and IoU prediction tasks separately. LADH consists of two core components: the Asymmetric Head and the Dual Head. The Asymmetric Head of LADH employs asymmetric multi-level compression to apply differentiated compression to features of different categories, thereby adapting to variations in the target complexity. The Dual Head is responsible for integrating the multi-scale feature outputs (P3–P5) from the Asymmetric Head and generating the final detection results.

LADH-Head uses depthwise separable convolution (DWConv) instead of standard convolution to avoid performance bottlenecks that shared feature layers may cause. Depthwise separable convolution decomposes traditional convolution into two independent operations: depthwise convolution and pointwise convolution. Specifically, pointwise convolution uses a 1×1 convolution kernel to fuse cross-channel information, adjusting only the number of channels while maintaining the spatial dimension of the feature map. This design further optimizes the model complexity. In the detection head design, the introduction of 3×3 depthwise separable convolution decouples the classification task from the bounding box regression task, effectively avoiding task interference caused by differences in the positive sample matching loss. Therefore, replacing the original decoupling head of YOLO11 with LADH-Head ensures the model’s lightweight nature while significantly improving the detection accuracy and computational efficiency through asymmetric feature processing, making it particularly suitable for track detection scenarios.

## 3. Experiments and Results Analysis

Current publicly available datasets focused on rail tracks primarily emphasize local features such as the track surface texture and localized defects, severely neglecting the holistic perspective essential for railway inspection tasks. This emphasis on local features results in significant discrepancies between existing datasets and real-world inspection scenarios, making it difficult to fully reflect the actual railway operational environment and thereby limiting the development of object detection algorithms tailored to this task. To address this issue, this study systematically constructed a dataset specifically tailored to the railway inspection context, thereby overcoming the limitations of existing datasets. This paper uses a self-built dataset to train and test the proposed model.

### 3.1. Experimental Datasets

Track image acquisition experiments were performed in a ballasted track field environment. The experimental site was selected as a standard rail ballasted track section. Fasteners and sleepers are crucial components of the track, serving as the primary connectors between the rails and the sleepers. Due to their unique shapes, this paper identifies and detects them by extracting the features of the fasteners and sleepers.

**Figure 9:**
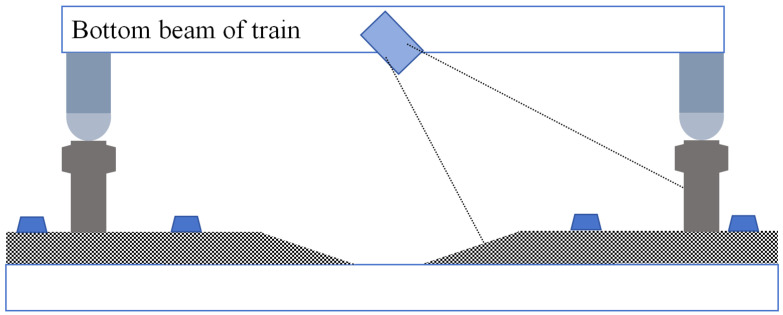
Data acquisition schematic.

Additionally, to enhance the practical usability of the dataset, this study specifically designed the image acquisition system to be mounted on the central section of the track inspection train, using a 45° installation angle to precisely target the bottom edge of the track for image capture. This structural design minimizes the inclusion of excessive external background environments, thereby maximizing the exclusion of external uncontrollable factors such as extreme weather conditions and sudden changes in lighting intensity that could affect the image data. This ensures that the captured track images effectively highlight the key visual features of the track itself. The camera’s installation position and framing angle are shown in Figure 9.

However, despite the rigorous image acquisition process, it is still challenging to fully account for the more complex real-world orbital detection environments, for example, dynamic changes in lighting conditions, the presence of random noise, fog caused by weather conditions, and varying degrees of obstruction by foreign objects between track structural components. Therefore, to enhance the completeness of the dataset, enable the model to learn more features, and improve the generalization ability of the deep learning model in complex scenarios, we employed image processing techniques to perform data augmentation on the original track image dataset, thereby striving to approximate and cover the scenarios that may be encountered in reality.

**Figure 10:**
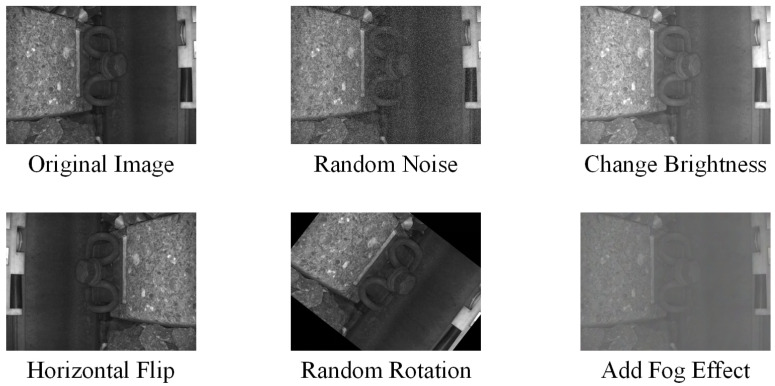
Raw data and data enrichment.

Specifically, five targeted image transformation methods shown in Figure 10 were implemented, and each augmentation method was parametrically controlled to ensure that the generated samples maintained the semantic authenticity of the original images while effectively extending the coverage of the data distribution, as described below:

- Geometric transformation: Performs a horizontal mirror flip of the original image.
- Luminance adjustment: Randomly adjust the image luminance value in the range of $\left[-30\%,+30\%\right]$ by linear transformation to simulate the track scene under different lighting conditions.
- Noise injection: A Gaussian noise model (sigma=0.05) was used to add random noise to the image to improve the robustness of the model to sensor noise.
- Random rotation: Rotate the image at a random angle to increase the spatial diversity of the samples.
- Atmospheric interference simulation: Based on the atmospheric scattering model [30], add the fogging effect of different concentrations to simulate the imaging characteristics under rainy and foggy weather conditions.

During the data collection experiments, a total of 566 valid track images were acquired, with an image resolution of 1408×1024 pixels. After the dataset augmentation, the LabelImg software was used to manually label the target area of the track images, draw bounding boxes around the track features, and assign categories. The annotated dataset consisted of 3396 images, including 3396 sleepers and 3396 fasteners. Finally, all the labeled images are divided into training sets (70%, 2377 images), validation sets (10%, 339 images), and test sets (20%, 680 images).

### 3.2. Experimental Environment and Parameter Configuration

The experiment was conducted in an environment with Windows 11, CUDA 12.6, Python 3.12.8, and Pytorch 2.5.1. The hardware configuration and training hyperparameters are presented in Table 2. During training, the input image size was uniformly adjusted to 640×640, and the L2 regularization term was used to penalize large weights to prevent overfitting.

### 3.3. Evaluation Indicators

To validate the performance of the algorithm proposed in this paper, we systematically evaluated the model using the following metrics: recall (R), precision (P), mean average precision (MAP), floating-point operations per second (FLOPS, denoted as F), parameter count, and frames per second (FPS), among others. Formulas for some of these metrics are provided below:

(6) $$P=\frac{T_{P}}{T_{P}+F_{P}}$$

(7) $$R=\frac{T_{P}}{T_{P}+F_{N}}$$

(8) $$MAP=\frac{\sum_{i=1}^{n}AP_{i}}{n}$$

(9) $$F=K^{2}\times C_{in}\times C_{out}\times H_{out}\times W_{out}$$

(10) $$Parameter=K^{2}\times C_{in}\times C_{out}+B$$

(11) $$FPS=\frac{1}{T}$$

In Equations (6) and (7), $T_{P}$ stands for true positive samples, $T_{P}$ stands for false positive samples, and $F_{N}$ stands for false negative samples; in Equation (8), $AP_{i}$ is the accuracy of the *i*th category; in Equations (9) and (10), *K* stands for the size of the convolutional kernel, $C_{in}$ is the number of channels in the input feature layer, $C_{out}$ is the number of channels in the output feature layer, $H_{out}$ is the height of the output feature layer, and $W_{out}$ is the width of the output feature layer; *B* stands for the bias term; in Equation (11), *T* is the time required for the model to infer a single sample (in *s*).

### 3.4. Convergence Test

We evaluate the convergence performance of the model by monitoring the trend of the loss function and optimizing the training strategy accordingly. Specifically, we focus on the change process of the following three types of loss functions:

- Bounding box loss, which is used to assess the regression effect of the target detection box;
- Distribution focal loss, which is used to optimize the distributional characteristics of the bounding box prediction;
- Classification loss, which is used to measure the classification performance of the model.

**Figure 11:**
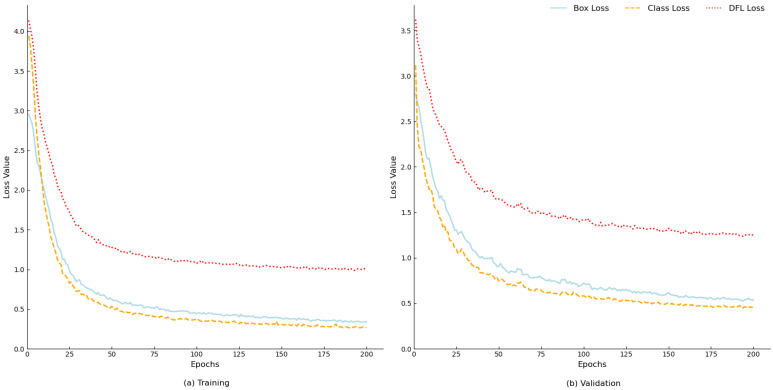
Model training loss value change curve.

The change curves of the above loss functions during the training process are shown in Figure 11.

Throughout the entire training process, as the number of training epochs increased, the total loss value of the model exhibited a monotonically decreasing trend, and the model displayed neither an obvious overfitting nor an underfitting phenomenon. When the number of training rounds reached 175, the loss curve gradually stabilized and entered a state of convergence. Subsequent performance evaluation and analysis could then be carried out after the loss function converged.

## 4. Analysis of Experimental Results

### 4.1. Ablation Experiment

To fully validate the effectiveness of the proposed improved method, ablation experiments are conducted on the datasets presented in this paper. Each set of experiments was conducted under the same environmental configuration and parameter settings. The results of the ablation experiments conducted using the six metrics of *MAP*50, *parameters*, model size, *FPS*, precision (*P*), and recall (*R*) for comparison are shown in Table 3.

The results show that the standard YOLO11 model achieves a detection MAP of 84.1%, a model size of 5.2 million parameters, and a detection speed of 118 frames per second. Improvements can be made to increase the model map to 84.9%, reduce the model size to 1.5 M, and increase the detection speed to 163 frames/s. The proposed method in this paper outperforms the YOLO11 model on all datasets, demonstrating its impressive performance in target detection and recognition.

As shown in Table 3, when using YOLO11n as the baseline model, each of the four improvements individually enhances the model’s lightweight nature. The model, which uses StarNet as the main feature extraction network module (YOLO11-Starnet), achieves a significant improvement in lightweight performance compared with the original model, with a 36.5% reduction in the model size and a 35.6% increase in the detection speed. However, this comes at the cost of a slight decrease in the average accuracy. Given the significant lightweighting achieved, this trade-off is acceptable, indicating that the element multiplication operations in StarNet can characterize object depth features with fewer parameters. The model incorporating the Detect-LADH detection head (YOLO11-Detect-LADH) shows a slight improvement in lightweight design and detection speed compared with the original model, indicating that Detect-LADH effectively balances accuracy and speed. The model incorporating the EPAN structure (YOLO11-EPAN) not only improves the model’s MAP but also achieves an unexpectedly high degree of lightweight optimization, indicating that EPAN can better fuse image scale features and demonstrate the effectiveness of its residual structure-like design. The model (YOLO11-C3K2-Light) that introduces the PConv module into the C3K2 module in the original neck model achieves a 0.7 percentage point improvement in average accuracy compared with the original model, while reducing the number of parameters and achieving a certain degree of lightweight optimization. When the C3K2-Light module is introduced into StarNet, Detect-LADH, and EPAN, it achieves varying degrees of accuracy improvement, all of which exceed the baseline model, indicating that the C3K2-Light module has good applicability. The lightweight track feature detection model (YOLO-LWTD) proposed in this paper performs well across all evaluation metrics, achieving an average precision, recall, and average precision that are 0.5, 2.0, and 0.8 percentage points higher than the original model, respectively. Meanwhile, the model size is only 1.5 MB, achieving a lightweight degree of 71.1%. The detection speed has improved by 45 frames per second, indicating that the improved model has a more concise and efficient network structure and can effectively enhance the model’s detection performance.

### 4.2. Comparison Test

To further validate the advantages of the improved YOLO11n for track feature recognition, a series of target detection models were selected for comparison tests. The constructed track dataset was used for training and evaluated on the test set. The test results are presented in Table 4.

From Table 4, the YOLO family of algorithms, which are also one-stage detection algorithms, have iteratively increased performances in terms of the average accuracy. However, YOLOv5, as a classic target detection algorithm, has a simple model structure that makes it have a lower model size and higher inference speed, but its accuracy is lower; YOLOv8, as an update of YOLOv5, has a greater improvement in accuracy; YOLOv9 has a smaller model size, but its detection speed is too low to apply to such a mobile detection task as track detection. YOLOv10 weighs the model size and detection accuracy; YOLO11, as a new generation of target detection algorithms, has been improved compared with the previous algorithms. YOLOv12 and YOLOv13 are iterative versions of YOLO11, with improvements in the average accuracy and lightweight performance, but neither is suitable for track detection scenarios. In this paper, the improved model we propose has satisfactory results in the precision rate, recall rate, average precision rate, model size, and inference speed. Specifically, relative to YOLOv5n, YOLOv8, YOLOv9, YOLOv10, and YOLO11, our model is 6.5, 2.8, 0.7, 0.9, and 0.5 percentage points higher regarding the precision rate; 7.2, 2.2, 7.0, 3.8, and 2.0 percentage points higher regarding the recall rate; and 7.4, 4.4, 3.3, 2.3, and 0.7 percentage points higher regarding the mean average precision rate, respectively. It is also 7% higher than YOLOv5 and YOLOv9 regarding the recall rate. Furthermore, the model size is lightened to 1.5M, which is significantly lower than the rest of the YOLO family of models, and its inference speed is 163 frames/s, which is much higher than the rest.

We designed and conducted a series of backbone network comparison experiments to evaluate the relative advantages of StarNet in terms of the model performance. We selected and included a variety of representative lightweight neural network modules as benchmark models, including FasterNet, ShuffleNetv2, EfficientNetv2, and MobileNetv3. The experimental results are shown in Table 5. The data shows that StarNet demonstrates significant advantages in comparative experiments. With a MAP50 accuracy of 83.1, it not only has the lowest number of parameters and smallest model size but also achieves the highest inference speed and lowest computational overhead. Compared with other networks, StarNet is not the most accurate network, but it performs better in terms of efficiency, speed, and resource utilization while maintaining a competitive accuracy, making it an efficient solution for track detection tasks.

To systematically evaluate the quality of the EPAN model in terms of its multi-scale feature fusion capabilities, BiFPN and SlimNeck were introduced as benchmarks for the comparative experimental analysis. The detailed comparison results are shown in Table 6. As can be seen from the experimental data in the table, EPAN demonstrates significant advantages in multiple key metrics: it achieves efficient inference with the fewest parameters, smallest model size, and lowest computational overhead, while also achieving the highest real-time performance and optimal recall rate. Although its MAP50 is slightly lower than SlimNeck, EPAN leads comprehensively in terms of accuracy and overall efficiency (e.g., FPS is 8.5% higher than PaNet and 33.3% higher than BiFPN), highlighting its superiority in balancing the accuracy, speed, and resource consumption.

### 4.3. Visual Analysis

**Figure 12:**
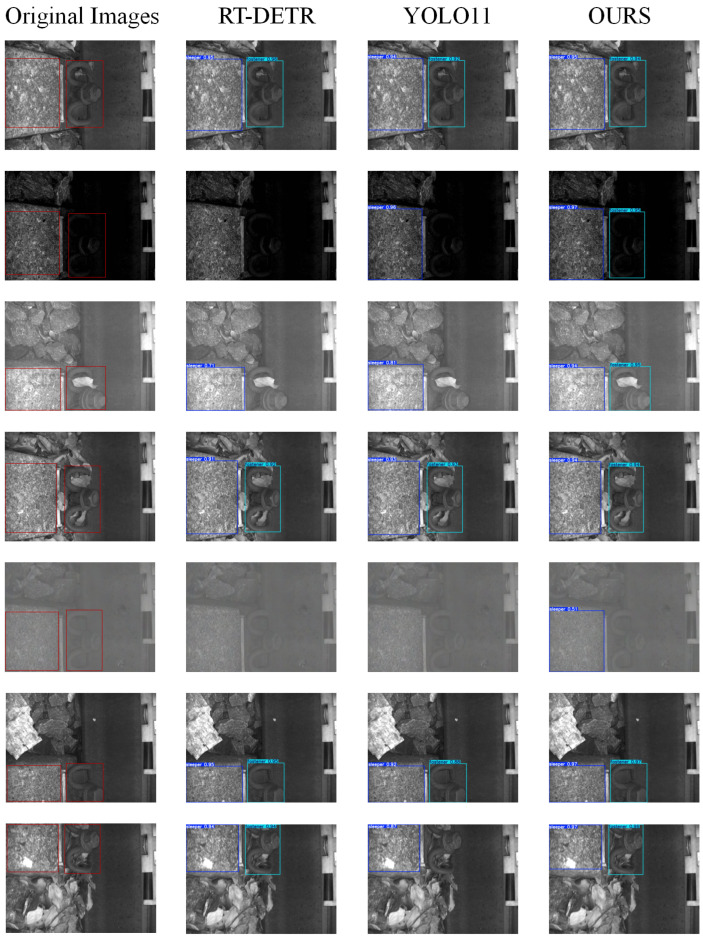
Test result chart.

The detection results obtained by training the track image dataset using the model proposed in this paper are shown in Figure 12.

As demonstrated by the seven visualization experiments, the YOLO-LWTD model proposed in this paper achieves good detection performance across various scenarios. In the ordinary scenes shown in the first column, there is little difference in the detection performance between all the models; the second and third columns represent low-light conditions at night and enhanced lighting during the day, respectively. The improved model focuses more closely on the detailed features of the track, whereas the original model is less sensitive to changes in lighting conditions. The fourth column introduces hazy weather conditions that may be encountered during track inspections, revealing that hazy weather significantly impacts the detection performance, with all models struggling to accurately identify objects. However, the proposed model still holds a certain advantage. The subsequent fifth and sixth columns further complicate the background information of the detection scenes. It can be seen that the improved model can better focus on the features of the target object, thereby achieving good detection results. Overall, the track detection model proposed in this paper can effectively focus on track features, and its lightweight structure will also demonstrate a more meaningful performance in practical applications.

**Figure 13:**
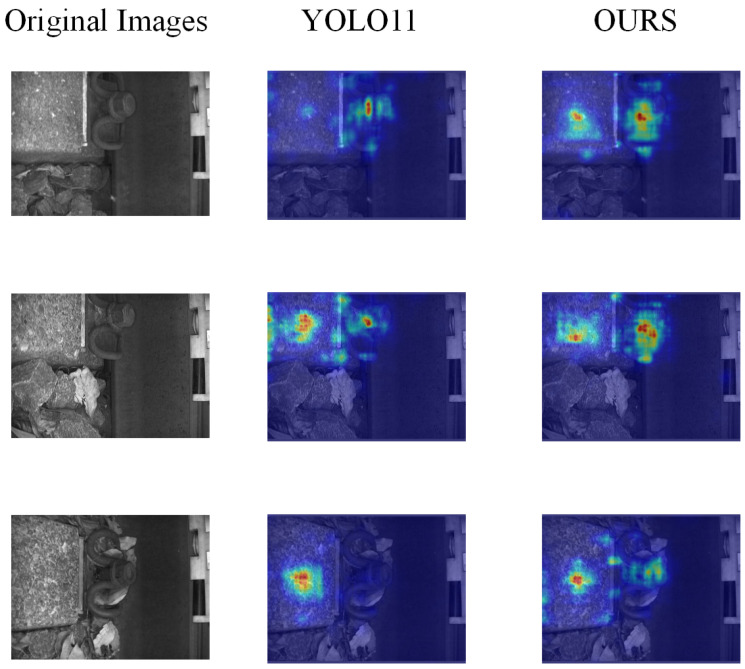
Heatmap results.

Heatmaps can more intuitively show the key location information learned by the model network. To more clearly evaluate the improved model, Grad-CAM was used to generate corresponding heatmaps, the results of which are shown in Figure 13.

As can be seen from Figure 13, the improved model exhibits significantly enhances the spatial focusing capability in the orbital feature region, with the CAM peak response region more concentrated around the geometric center of the orbital structure. This phenomenon intuitively confirms the effectiveness of the model optimization.

### 4.4. Deployment Testing

**Figure 14:**
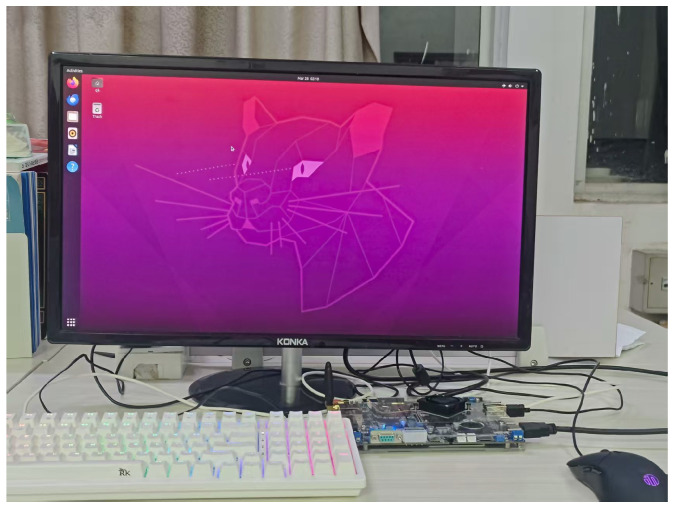
Experimental platform.

We built the deployment platform shown in Figure 14 based on Rockchip RK3588. RK3588 uses a high-performance processor that integrates quad-core Cortex-A76 and quad-core Cortex-A55 architectures, equipped with an ARM Mali-G610 MC4 GPU, 16 GB LPDDR4X memory, and 64 GB eMMC storage, and supports multiple operating systems, including Linux.

Experiments were conducted on a mobile terminal platform using the Ubuntu 22.04 to perform Int8 quantization on the model. The results showed that the original model size was 5.2 MB with an FPS of 52, while the improved model size was 1.4 MB with an FPS of 69. The lightweight design of the improved model was proven to be effective and suitable for deployment on mobile platforms, providing a highly energy-efficient solution for lightweight track detection tasks targeting embedded deployment or real-time processing.

## 5. Summary and Future Work

### 5.1. Summary

In this study, the network structure of YOLO11n is enhanced by introducing StarNet into the backbone network, resulting in a lower model size and a significantly higher detection speed. This makes it suitable for multi-threaded work. A work structure is introduced in the neck to improve the feature fusion effectiveness of the enhanced model, and some C3K2-Light attention modules are added to improve the model’s lightness. Finally, the model is further improved by Detect-LADH to further improve the detection effect of the model.

The results show that the proposed improved YOLO11n can improve the detection accuracy of the model for track features, which is 0.5, 2.0, and 0.8 percentage points higher than the original model in terms of the mean values of precision, recall, and average precision, respectively; the inference speed is 163 frames/s, which is 38.1% higher than that of the original model, and the size of the model is only 1.5 MB, with a lightweight degree of 71.1%. The performance of the improved model is more balanced in terms of the detection accuracy and degree of lightweight, with a lower model size and significantly higher detection speed. This makes it suitable for multi-threaded, real-time track detection processing tasks and deployment on mobile devices.

### 5.2. Future Work

This study conducted experimental validation based on a diverse dataset of track images. However, it is worth noting that the current dataset still has limitations in covering all possible track scenarios. Due to the lack of a widely recognized public benchmark dataset specifically designed for track feature detection within the industry, this research faces certain challenges regarding the comprehensiveness and universality of the dataset. This limitation also represents one of the key issues that future research needs to address.

Looking ahead, our work will focus on the following directions: (1) continuously expanding the scale and diversity of datasets while building an open-access benchmark dataset for track features to foster collaborative development in this field; (2) applying more rigorous statistical methods, such as cross-validation, to further validate the model generalization capabilities on larger datasets; (3) addressing highly heterogeneous challenges in railway inspection operations—such as dynamic environmental changes, extreme weather, and complex terrain—by prioritizing research on robust perception and intelligent recognition technologies for complex, multi-variable scenarios (e.g., low-light conditions, rain/fog interference, track debris, and high-speed moving perspectives); (4) explore the deep integration and collaborative analysis of multi-source heterogeneous sensing methods—including high-resolution machine vision, 3D laser scanning, multispectral/infrared imaging, ground-penetrating radar, acoustic detection, and inertial measurement units—to build next-generation intelligent track inspection systems characterized by high precision, real-time capability, and reliability.

## Figures and Tables

**Table 1:** YOLO11 model parametric quantities and calculations.

Model	FLOPs/B	Param/M
YOLO11n	6.5	2.6
YOLO11s	21.5	9.4
YOLO11m	68.0	20.1
YOLO11l	86.9	25.3
YOLO11x	194.9	56.9

**Table 2:** Experimental environment parameters.

Parameter Name	Parameter Information
CPU	Intel® Core i5-13490F (Santa Clara, CA, USA)
Memory	32 GB
Display card	NVIDIA GeForce RTX 4070 GPU (Santa Clara, CA, USA)
Epoch	200
Batch size	16
Image size	640 × 640
Model size	Depth: 0.25, Width: 0.25
Weight decay	0.0005
Initial learning rate	0.01
Optimizer	Adam

**Table 3:** Table of ablation experiments.

Base	StarNet	Detect-LADH	EPAN	C3K2-Light	MAP50 (%)	Parameters	Size (M)	FPS	P (%)	R (%)
✓					84.1	2,582,542	5.2	118	97.7	71.0
✓	✓				83.1	1,598,406	3.3	160	96.5	70.8
✓		✓			83.6	2,281,742	4.7	120	97.8	69.9
✓			✓		85	1,788,782	3.7	128	98.3	73.2
✓				✓	84.8	2,431,646	4.9	118	96.5	72.6
✓	✓			✓	85.9	1,447,510	3	152	98.8	74.3
✓		✓		✓	84.3	2,130,846	4.4	117	97.2	73.3
✓			✓	✓	85.5	1,768,318	3.7	126	97.9	73.8
✓	✓	✓	✓	✓	84.9	620,598	1.5	163	98.2	73.0

**Table 4:** Comparative test table.

Model	MAP50 (%)	Parameters	Size (M)	FPS	P (%)	R (%)	GFLOPs
YOLOv5 [31]	77.5	2,182,054	4.4	138	91.7	65.8	5.8
YOLOv8 [32]	80.5	2,684,785	5.4	133	95.4	70.8	6.8
YOLOv9 [33]	81.6	1,756,950	4	67	97.5	66	6.5
YOLOv10 [34]	82.6	2,695,196	5.5	104	97.3	69.2	8.2
YOLO11	84.1	2,582,542	5.2	118	97.7	71.0	6.3
YOLOv12 [35]	84.5	2,557,118	5.3	72	98.3	71.9	6.3
YOLOv13 [36]	83.7	2,448,285	5.2	52	98.8	66.4	6.2
RTDETR (ResNet50) [37]	84	41,938,794	82	10	88.9	77.9	125
Ours	84.9	620,598	1.5	163	98.2	73.0	1.7

**Table 5:** Results of the backbone network comparison experiment.

Backbone	MAP50 (%)	Parameters	Size (M)	FPS	P (%)	R (%)	GFLOPs
StarNet	83.1	1,598,406	3.3	160	96.5	70.8	3.7
FasterNet [38]	82.9	2,378,314	5.1	128	93.1	69.1	5.5
ShuffleNetv2 [39]	74.9	1,705,318	3.6	102	94.2	65	4.1
EfficientNetv2 [40]	81.7	2,086,978	4.4	96	97.1	69	5.2
MobileNetv3 [41]	83.3	2,144,716	4.4	114	96.2	73.1	3.8

**Table 6:** Results of the neck network comparison experiment.

Neck	MAP50 (%)	Parameters	Size (M)	FPS	P (%)	R (%)	GFLOPs
EPAN	85	1,788,782	3.7	128	98.3	73.2	5.2
PaNet [42]	84.1	2,582,542	5.2	118	97.7	71.0	6.3
BiFPN [43]	84.7	2,670,538	5.4	96	98.9	68.4	7
SlimNeck [44]	85.2	2,731,678	5.6	105	94.6	70	6.3

## Data Availability

The original contributions presented in this study are included in the article. Further inquiries can be directed to the corresponding author.
